# HMGB1 and thrombin mediate the blood-brain barrier dysfunction acting as biomarkers of neuroinflammation and progression to neurodegeneration in Alzheimer’s disease

**DOI:** 10.1186/s12974-016-0670-z

**Published:** 2016-08-24

**Authors:** Barry W. Festoff, Ravi K. Sajja, Patrick van Dreden, Luca Cucullo

**Affiliations:** 1 pHLOGISTIX LLC, 4220 Shawnee Mission Parkway, Fairway, KS 66205 USA; 2 Department of Neurology, University of Kansas Medical Center, 3901 Rainbow Blvd, Kansas City, KS 66160 USA; 3 Department of Pharmaceutical Sciences, Texas Tech University Health Sciences Center, 1300 S. Coulter Street, Amarillo, TX 79106 USA; 4 Clinical Research Department, R&D, Diagnostica Stago, Gennevilliers, France

**Keywords:** Biomarkers, Blood-brain barrier, Clinical, DAMPS, HMGB1, Neuroinflammation, Neurodegeneration, Permeability, Thrombin

## Abstract

**Background:**

The blood-brain barrier (BBB) dysfunction represents an early feature of Alzheimer’s disease (AD) that precedes the hallmarks of amyloid beta (amyloid β) plaque deposition and neuronal neurofibrillary tangle (NFT) formation. A damaged BBB correlates directly with neuroinflammation involving microglial activation and reactive astrogliosis, which is associated with increased expression and/or release of high-mobility group box protein 1 (HMGB1) and thrombin. However, the link between the presence of these molecules, BBB damage, and progression to neurodegeneration in AD is still elusive. Therefore, we aimed to profile and validate non-invasive clinical biomarkers of BBB dysfunction and neuroinflammation to assess the progression to neurodegeneration in mild cognitive impairment (MCI) and AD patients.

**Methods:**

We determined the serum levels of various proinflammatory damage-associated molecules in aged control subjects and patients with MCI or AD using validated ELISA kits. We then assessed the specific and direct effects of such molecules on BBB integrity in vitro using human primary brain microvascular endothelial cells or a cell line.

**Results:**

We observed a significant increase in serum HMGB1 and soluble receptor for advanced glycation end products (sRAGE) that correlated well with amyloid beta levels in AD patients (vs. control subjects). Interestingly, serum HMGB1 levels were significantly elevated in MCI patients compared to controls or AD patients. In addition, as a marker of BBB damage, soluble thrombomodulin (sTM) antigen, and activity were significantly (and distinctly) increased in MCI and AD patients. Direct in vitro BBB integrity assessment further revealed a significant and concentration-dependent increase in paracellular permeability to dextrans by HMGB1 or α-thrombin, possibly through disruption of zona occludins-1 bands. Pre-treatment with anti-HMGB1 monoclonal antibody blocked HMGB1 effects and leaving BBB integrity intact.

**Conclusions:**

Our current studies indicate that thrombin and HMGB1 are causal proximate proinflammatory mediators of BBB dysfunction, while sTM levels may indicate BBB endothelial damage; HMGB1 and sRAGE might serve as clinical biomarkers for progression and/or therapeutic efficacy along the AD spectrum.

## Background

Despite overarching evidence, the amyloid hypothesis in Alzheimer’s disease (AD) first elaborated in 1991, has yet to provide positive outcomes notwithstanding the billions spent on clinical trials [1]. The neuropathological hallmarks of AD are extracellular amyloid beta (Aβ)/neuritic plaques and intracellular neurofibrillary tangle (NFT) formation [2]. In association with these hallmarks, soluble Aβ levels increase in the blood, both in AD patients and transgenic mouse models [3–5], while the brain amyloid β aggregates promote a neuroinflammatory response mediated by activated microglia, astrocytes, and microvascular endothelial cells (ECs) [6, 7]. Since late-onset AD (LOAD) is not associated with such manifestations information from AD, transgenic animal models cannot be fully extrapolated to human AD pathology. Furthermore, microglial activation and other aspects of neuroinflammation involving oxidative stress (reactive oxygen species (ROS), nitric oxide (NO)) actually precede neuronal damage [8, 9], prior to AD histopathologic lesions. Moreover, a critical characteristic of neuroinflammation is the disruption of the blood-brain barrier (BBB) that extends beyond the tissue or cellular pathophysiology of endothelial cell (EC) dysfunction to the neurovascular unit (NVU), including astrocytic end-feet, microglia, neurons, and pericytes [10–12]. Recent National Institutes of Health (NIH) workshops have emphasized key areas that must be focused on as it relates to AD neuroinflammation research involving the BBB: (1) transport of Aβ and other macromolecules in and out of the brain, i.e., *permeability*; (2) BBB as both the *source* and *target* of inflammatory factors; and (3) effects of oxidative stress, ROS, and NO on BBB. Besides astrogliosis, activation and transmigration of blood-borne substances and circulating immune cells into the CNS is a less studied and underappreciated area in AD research [13–16]. The precise molecular factors governing the initial BBB damage leading to neurodegeneration, in general, and AD, in particular, are not well understood.

Thrombin and high-mobility group box protein 1 (HMGB1) are key molecules of two most potent host defense systems that converge on the innate immune system, coagulation, and inflammation. We postulated that they may play significant roles in the BBB disruption since both are proinflammatory and both are known to disrupt vascular barriers in other tissues [17–20]. Thrombin is a proinflammatory serine protease that is well known for its essential role as the ultimate protease in the coagulation pathway. HMGB1 is a non-histone nuclear protein with dual functions depending on localization. Within the cells, it is localized primarily to the nucleus where it binds DNA and plays a role in transcriptional regulation [21]. However, extracellular HMGB1 serves as a proinflammatory cytokine and is a late mediator of sepsis [22]. Beyond infections, HMGB1 has pathogenic roles during trauma and sterile inflammation, such as systemic inflammatory response syndrome (SIRS), where elevated levels in sera orchestrate key events including leukocyte recruitment and white blood cell (WBC) induction to secrete inflammatory cytokines [23, 24].

Relevant to our studies, HMGB1 impairs memory behavior in mice that is mediated via Toll-like receptor 4 (TLR4) and the receptor for advanced end product glycation (RAGE) [25]. These pre-clinical data correlate with clinical studies showing that sepsis survivors have permanent cognitive deficits [26] and that these may also be mediated via HMGB1, but the precise mechanism remains unknown. HMGB1 and another alarmin, S100B, along with Aβ, are now considered as three significant damage or danger-associated molecular patterns (DAMPs) that “fan the flame” [27] of neuroinflammation in AD [28]. How they might do this is currently unknown. As an approach to this problem, we first measured levels of these DAMPs in mild cognitive impairment (MCI), AD, and normal aged subjects and then used pure proteins in the range of these levels to perturb human BBB function in vitro.

## Methods

### Human subjects and specimen collection

The human study was approved by the institutional review board at the University of Kansas Medical Center (KUMC) and was performed in compliance with the Helsinki Declaration. All subjects were recruited from the KU Alzheimer Disease Center (ADC) following a written informed consent, and all demographic information was de-identified. Subjects in the AD, MCI, and aged control cohorts met the criteria as outlined in the 2011–2012 NIA/Alzheimer’s Association Guidelines for AD by McKhann et al. [29] and for MCI by Albert et al. [30]. Furthermore, all subjects were screened by clinical dementia rating (CDR) and neuropsychological testing as defined by the Uniform Data Set of the Alzheimer Disease Centers and underwent a consensus review. MCI patients were further classified as “MCI due to AD” if another more likely cause was not identified. Our MCI cohort patients were all designated MCI due to AD.

Venous blood collection was performed with BD Vaccutainer® blood collection tubes and centrifuged. The serum samples were aliquoted and stored at −80 °C until analysis. Demographic information for these patient groups is shown in Table 1.

**Table 1:** Human subject demographics (gender and age were shown for each subject in AD, MCI, and control cohorts at the time of blood collection)

Patient ID	Gender	Age at draw	Dx at draw
1	F	80	AD
2	F	77	AD
3	F	81	AD
4	F	60	AD
5	M	63	AD
6	M	76	AD
7	M	73	AD
8	M	81	AD
9	M	67	AD
10	M	70	AD
11	M	84	AD
12	M	88	AD
1	F	67	MCI
2	M	88	MCI
3	M	76	MCI
4	M	74	MCI
5	M	77	MCI
6	M	64	MCI
7	M	72	MCI
8	M	84	MCI
9	M	68	MCI
10	M	72	MCI
11	M	75	MCI
12	M	65	MCI
1	F	77	NL
2	F	76	NL
3	F	78	NL
4	F	66	NL
5	F	69	NL
6	F	74	NL
7	F	81	NL
8	F	75	NL
9	F	72	NL
10	F	66	NL
11	M	69	NL
12	M	76	NL

### Cell culture

The human cerebromicrovascular endothelial cell line (hCMEC/D3) was a gift from Dr. P.O. Couraud (INSERM, France). This cell line has been widely used as a representative model of human BBB in vitro for mechanistic studies involving the molecular regulation of BBB integrity [31]. Cells between passages 28 and 31 were cultured in HEPES-buffered EBM-2 media supplemented with growth factors and antibiotics and maintained at 37 °C with 5 % CO_2_ exposure. In preliminary experiments, primary human brain microvascular endothelial cells (HBMEC) were cultured in Dr. KY Kim’s laboratory (KUMC) as described previously [32].

### Analysis of serum biomarkers

Human serum samples were thawed on ice and analyzed by enzyme-linked immunosorbent assay (ELISA) for quantification of HMGB1 (Novatein Biosciences, Woburn, MA), s100β (EMD Millipore, Billerica, MA), amyloid β (aa 1-42), and soluble receptor for advanced glycation end products (sRAGE) from R&D Systems (Minneapolis, MN, USA), according to the manufacturers’ instructions. Soluble thrombomodulin (sTM) antigen (TMa) levels were assayed by kit (Stago, Gennevilliers, France), and sTM activity was measured as described previously [33].

### BBB integrity assessment

Cells were seeded on Corning Transwell® polyester membrane inserts (12-well, 0.4 μm pore size) as previously described [31]. Monolayer integrity and morphology were periodically assessed by transendothelial electrical resistance (TEER; Ω cm^2^) and phase contrast microscopy, respectively. Cells were maintained in treatment media (EMB-2 supplemented with 10 mM HEPES, antibiotics, and 1 % human serum) overnight prior to treatment with human recombinant HMGB1 protein (rHMGB1) with endotoxin levels lower than 0.1 EU per microgram of the protein (R&D Systems, Minneapolis, MN, USA). Following 3 h of treatment with rHMGB1 (5, 10, and 50 ng/mL), α-thrombin (0.5, 1, and 5 nM) or control media, inserts were transferred to new 12-well plates and transport buffer added (pre-warmed HBSS buffer with antibiotics and 0.5 % BSA) to the apical (500 μL) and basal compartment (1000 μL). After incubation for 20 min at 37 °C under 5 % CO_2_ exposure, a mixture of florescent dextrans of increasing molecular sizes (FITC-4 kDa, 10 mg/mL; Cascade Blue 10 kDa, 5 mg/mL; and rhodamine B isothiocyanate (RITC)-70 kDa, 10 mg/mL) was added to the apical compartment. Dextran permeability (luminal to abluminal flux) was measured at 30 min following the addition of dextrans. Apparent permeability coefficients (Pe; centimeters per second) were calculated and expressed as percent control [31]. For pharmacological inhibition (validation) studies, cells were co-incubated with anti-HMGB1 antibody (gift from Dr. Michael Bustin, NIH) or non-specific control antibody for the indicated duration.

### Immunofluorescence analysis

Immunocytochemistry (ICH) for zona occludin-1 (ZO-1) was performed using primary anti-ZO-1 antibody (Zymed Laboratories, San Francisco, CA), Alexa Fluor 488-conjugated secondary antibody and 4,6-diamidino-2-phenylindole (DAPI; Molecular Probes, Eugene, OR) as described in [32].

### Statistical analysis

Data were expressed as mean ± SEM or SD. Data were analyzed by one-way analysis of variance (ANOVA) followed by Dunnett’s or Tukey’s post hoc multiple comparison tests using GraphPad Prism Software Inc. (La Jolla, CA, USA). *P* value was set to less than 0.05 for statistical significance.

## Results

### Serum profile of proinflammatory “damage-associated” factors in AD, MCI, and age-matched normal subjects

Part of the criteria for AD and MCI due to AD infers that other more likely diagnoses are less likely than AD. Although it does not totally eliminate, it clearly should limit contamination by other diagnoses such as vascular dementia, Parkinson’s disease with dementia, and primary systemic inflammatory diseases.

**Fig. 1:**
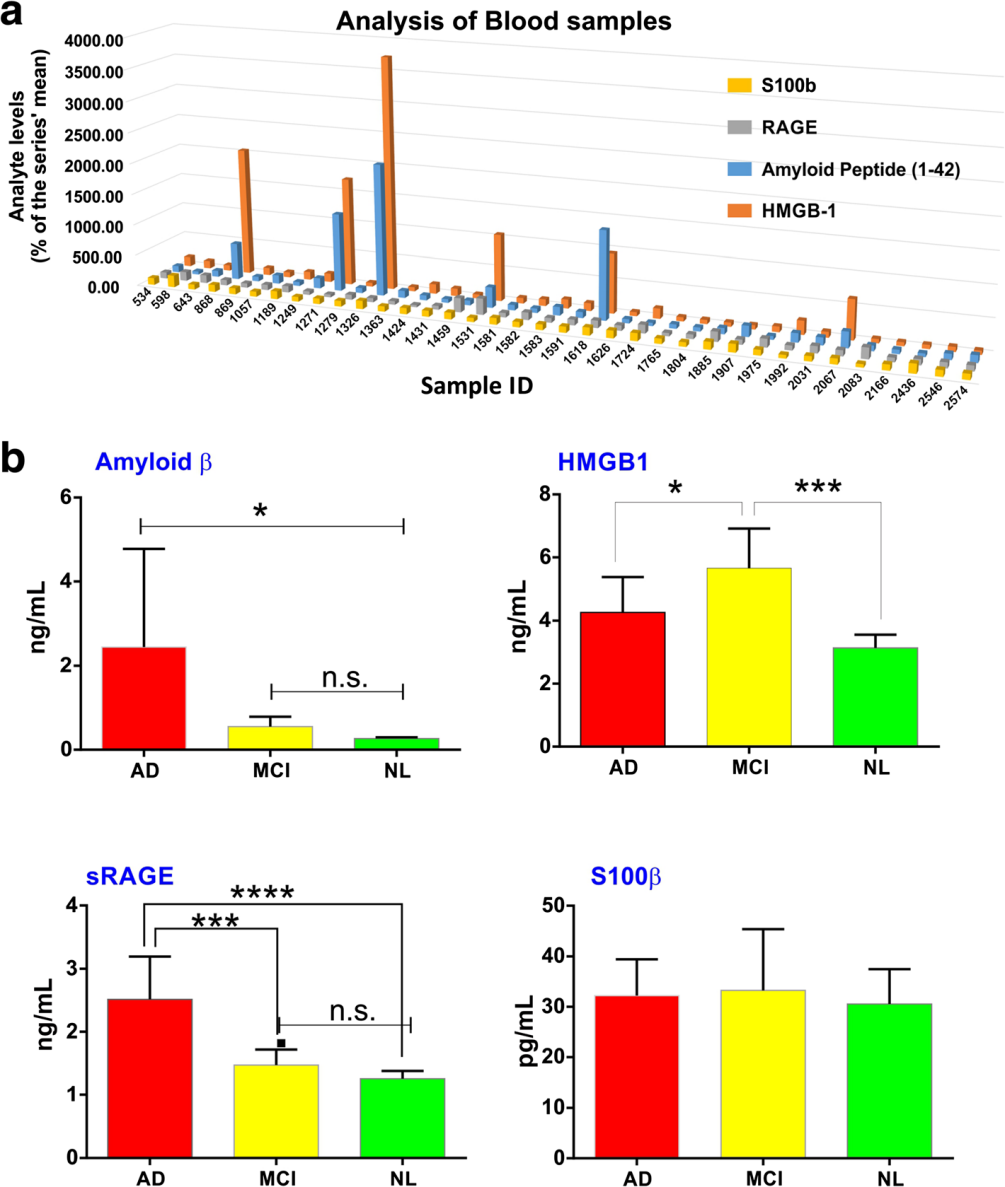
Serum profiling of clinical biomarkers of neuroinflammation to predict the conversion (continuum) of MCI to AD. **a** The serum levels of Aβ, HMGB1, sRAGE, and s100β are shown individually for each subject belonging to age-matched control (NL), MCI, or AD cohorts to facilitate the correlation of these markers. **b** Serum levels of each DAMP expressed as mean (±SD) for each cohort. *p < 0.05 and ***p < 0.01. *n.s.* not significant

Levels of various proinflammatory markers including Aβ, sRAGE, HMGB1, and S100β in serum samples derived from control subjects (NL), AD, or MCI patients (see Table 1 for demographic information) were measured by ELISA. As shown in Fig. 1, Aβ levels were significantly higher in AD patients when compared to MCI and NL. No statistically significant differences were noted between MCI and NL groups. An unprecedented pathophysiological parallelism between Aβ and sRAGE (the soluble, circulating form of RAGE) [34, 35] levels was also noted, as sRAGE was equally increased in the plasma of AD patients vs. MCI and controls (Fig. 1). In contrast, parallel measurements of HMGB1 in the MCI cohort was significantly higher than the levels measured in AD and control subjects. A side-by-side comparison between AD and NL revealed a higher trend for HMGB1 levels in AD patients although the results were not statistically significant. No significant differences between the three groups were observed with respect to S100β levels.

The critical message from these results is that these proinflammatory damage-associated molecules [28] (with the exception of s100β) show specific patterns of expression in relationship to the continuum from normal aging→MCI→AD. This strongly suggests that HMGB1 and/or sRAGE in addition to Aβ could have a potential use as prognostic/diagnostic markers for AD and MCI.

### Serum expression/activity of soluble thrombomodulin in AD, MCI, and control subjects

**Fig. 2:**
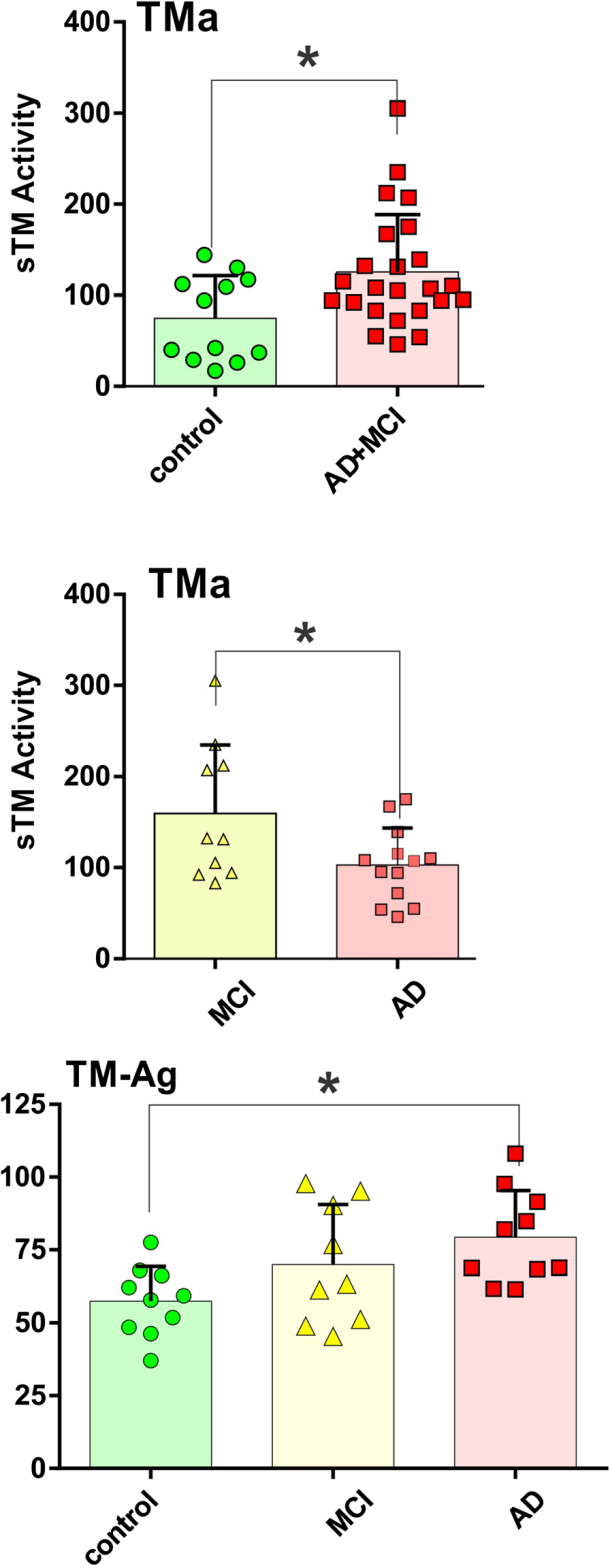
Soluble thrombomodulin activity (TMa) and expression (TM-Ag) in AD, MCI, and control subject sera. TMa and TM-Ag were assayed by kits as described in the “Methods” section. *Top panel* represents the combined TMa in MCI and AD patients above age-matched controls. *Middle panel* shows the differences in TMa between MCI and AD patients. *Bottom panel* represents the serum levels of TM-Ag in control, MCI, and AD subjects. Data were expressed as mean ± SEM and *p < 0.05

Additionally, in our efforts to develop a validated, non-invasive biomarker for BBB EC damage in AD, we measured both sTM antigen (TM-Ag) and TM activity (TMa; which converts protein C to activated PC; see “Methods” section). As shown in Fig. 2, we found significant increase in TMa above age-matched controls when MCI and AD levels were grouped (upper panel). We also observed that MCI levels were, in fact, statistically higher than those measured in AD patients (middle panel). However, as shown in Fig. 2 (bottom panel), the following relationship was found for TM Ag: AD>MCI>control (lower panel) with a significant increase in AD vs. control subjects. Similar to remitting relapsing multiple sclerosis (RRMS) patients [36], increased levels of sTM occur in sera of AD and MCI patients as compared with controls (Fig. 2).

### Effects of proinflammatory HMGB1 and thrombin on BBB permeability in vitro

**Fig. 3:**
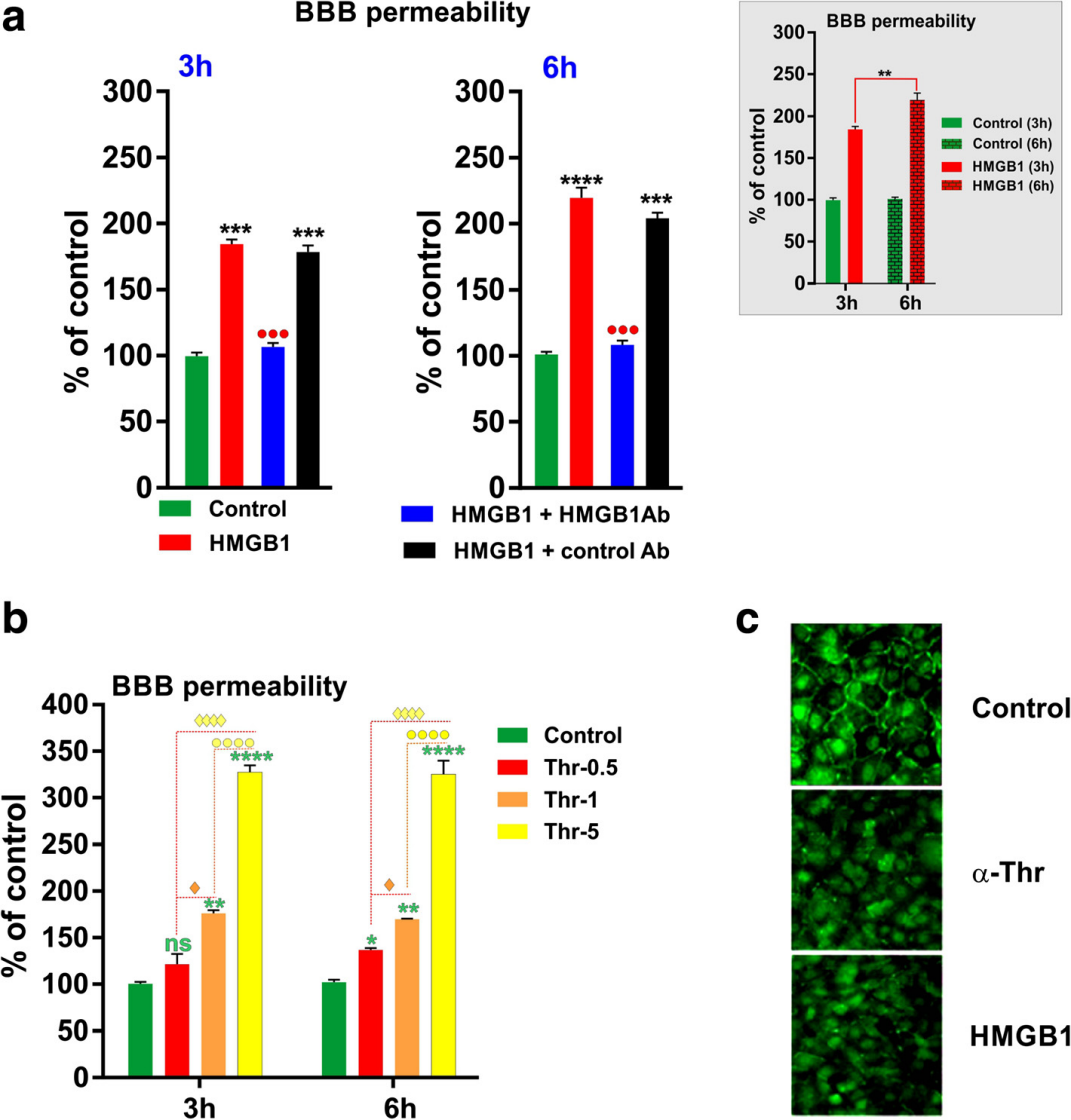
HMGB1 and thrombin impair BBB integrity. HBMEC monolayers were incubated with HMGB1 (10 ng/ml) and thrombin (Thr, 0.5, 1, or 5 nM) for 3 or 6 h, and BBB integrity was assessed by paracellular permeability to FITC-dextran. **a** HMGB1 increases luminal to abluminal dextran flux across HBMECs that was reversed by anti-HMGB1 antibody (HMGB1 Ab, 10 ng/ml). The time-dependent increase in HMGB1-induced BBB permeability was shown in the *inset*. **b** Exposure to thrombin (3 or 6 h) dose-dependently increases BBB permeability to dextran in HBMECs. **c** HMGB1 and thrombin disrupt ZO-1 linearity at cell-cell junctions. *p < 0.05, **p < 0.01, and ***p < 0.001 vs. control; ●●●*p* < 0.01 vs. HMGB1 or HMGB1 + control Ab. *N* = 5/condition

To directly assess microvascular damage associated with elevated levels of these proinflammatory DAMPs in AD and MCI patients, we next determined whether HMGB1 and/or the proinflammatory coagulation protease, thrombin, players in vascular barrier dysfunction in other tissues could disrupt BBB integrity. As shown in Fig. 3a, one-way ANOVA revealed that pre-treatment with recombinant human HMGB1 (10 ng/mL) protein for 3 or 6 h significantly increased BBB apical to basal permeability to FITC dextran across the HBMEC monolayers (*p* < 0.001 vs. control). We also observed that the magnitude of dextran flux was more pronounced with the duration of exposure to HMGB1 (*p* < 0.01, 6 vs. 3 h; see inset in Fig. 3a). Importantly, HMGB1-induced increase in BBB paracellular dextran permeability could be significantly and specifically blocked by neutralizing antibody against HMGB1 [37], whereas control (pre-immune) antibody failed to suppress HMGB-1-induced BBB disruption at either incubation time (Fig. 3a). Intriguingly, our preliminary data also indicated that a specific fragment of rTM, called TM-D1, the C-type lectin-like domain [38], can effectively block rHMGB1 activity, thus protecting the BBB against this DAMP (data not shown).

Similarly, when we tested the effects of thrombin on BBB integrity, we observed a significant and concentration-dependent increase in FITC-dextran permeability across the HBMEC monolayers (Fig. 3b). For instance, higher thrombin concentrations (5 nM) more potently induced BBB disruption (*p* < 0.0001 vs. control) when compared to dextran permeability resulting from exposure to 0.5 or 1 nM (*p* < 0.001). However, we did not observe a further elevation in thrombin-induced BBB dextran flux with longer exposure time (3 vs. 6 h), suggesting a maximum and sustained response following 3 h of exposure. The negative impact of HMGB1 and thrombin on BBB integrity is further confirmed by corresponding immunostaining of HBMECs that demonstrated a significant downregulation of ZO-1 expression at intercellular tight junctions (TJs) (Fig. 3c).

**Fig. 4:**
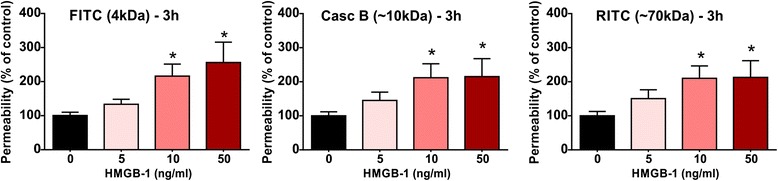
HMGB1 increases BBB permeability to dextrans of various sizes. HCMEC/D3 cells in the transwells were incubated with HMGB1 (5, 10, or 50 ng/ml) for 3 h and the apical to basal flux of a mixture of florescent-labeled dextrans of different sizes (FITC-4 kDa; Cascade Blue 10 kDa; and RITC-70 kDa) was assayed following 30 min after the addition of dextrans. (*p < 0.05 vs. control and *N* = 5/condition)

We next determined the extent of BBB disruption following exposure to HMGB1 by assessing the permeability to florescent dextrans of increasing molecular sizes (4–70 kDa) across the hCMEC/D3 monolayers (see “Methods” section). As shown in Fig. 4, HMGB1 exposure for 3 h significantly and dose-dependently increased the luminal to abluminal flux of all labeled dextrans across the hCMEC/D3 monolayers in a size-selective manner. The main effect of the treatment on 4-, 10-, and 70-kDa dextran permeability was significant, as indicated by one-way ANOVA. Further analyses by post hoc tests revealed that high concentrations of HMGB1 (10 and 50 ng/mL) significantly increased apical to basal permeability of each dextran by 2.0–2.5-fold, as compared to aged control group (*p* < 0.05 and *p* < 0.05, respectively; Fig. 4). Although, HMGB1 at low concentration (5 ng/mL) showed a marked increase in BBB permeability to all dextrans, this effect was non-significant. In addition, we observed a ceiling effect on BBB permeability to 10 and 70 kDa dextrans at HMGB1 (10 ng/ml; Fig. 4), as further increase in concentration to 50 ng/mL did not elevate the permeability to these dextrans.

## Discussion

Although rigorous methods are used by the KU ADC to enroll subjects in AD, MCI, MCI due to AD and normal cohorts, one can never fully eliminate other systemic illnesses that escaped notice. Such recruitment methods are part of the approach that our colleagues at the KU ADC take, quite similar to methods taken at other institutional ADCs. In this regard, our current results indicate that three potent proinflammatory and damage/danger-associated macromolecules are elevated in MCI and AD sera: Aβ, sRAGE, and HMGB1. They further suggest that one or more may herald the conversion of MCI to AD (Fig. 1). Furthermore, our BBB in vitro data show that one of these, HMGB1, along with the proinflammatory coagulation protease, thrombin, are proximate mediators of BBB dysfunction. With our present results, we conclude that HMGB1 might be a better clinical biomarker, along with sRAGE and Aβ, to predict the course of neurodegeneration in AD subjects. In contrast, although it also activates RAGE [39], from our results, S100B does not appear to have applicability as a diagnostic or prognostic marker of neurodegeneration in MCI and AD patients.

Considerable evidence indicates that blood levels of sTM and von Willebrand factor (vWf) can serve as surrogate biomarkers for diffuse microvascular damage [39]. We had previously found that sTM plasma levels were higher in RRMS patients than in patients with systemic lupus erythematosus with diffuse microvascular inflammation, indicating its potential utility as a biomarker for BBB damage [36]. Relevant to our current results, others had suggested that sTM, along with vWf, might be a good biomarker for AD-related microvascular damage [40]. In our present studies, soluble forms of the anticoagulant/anti-inflammatory TM [38, 41] might also serve as a biomarker for microvascular damage of the BBB in AD. This is strongly suggested by the pattern of expression for the sTM antigen which progressively increases from controls to ↑MCI to ↑AD although only the difference between control and AD was statistically significant at this stage. Perhaps future studies involving a larger cohort of patients will be able to provide additional statistical power to the analysis. When data were aggregated, TMa was greater in disease (MCI + AD) than aged-matched controls (see Fig. 2).

As with other vascular barriers [17, 18], human alpha thrombin significantly compromised BBB integrity in vitro at nanomolar concentrations (Fig. 3b), orders of magnitude less than the circulating prothrombin levels in the blood [38]. In an attempt to explain the thrombin’s effect on brain edema, Guan and colleagues injected thrombin stereotactically into the rat caudate nuclei and found a marked extravasation of Evans Blue dye, suggesting BBB disruption [42]. These authors also demonstrated that exposure to thrombin upregulated the endothelial expression of matrix metalloproteinase-2 possibly through the activation of protease-activated receptor 1 (PAR1). In similar direct experiments using different vascular barrier models, Garcia’s group and others showed that thrombin mediated barrier disruption, indeed, via a PAR1 mechanism [17, 18, 43]. Although, we have no current direct evidence that PAR1 mediated the BBB disruption effects of thrombin, the nanomolar concentrations are in agreement with mediation via PAR1. However, our preliminary experiments suggested that the BBB disruptive effects of thrombin are a direct measure of its proteolytic activity, since recombinant hirudin, a specific thrombin inhibitor, inhibited the effects of thrombin (data not shown). Further indicating thrombin was likely interacting with a protease-activated receptor (PAR) on the EC surface, an rTM fragment that binds both thrombin and PC (TMD23) also blocked thrombin’s effects on BBB permeability (not shown).

The proinflammatory DAMP, HMGB1, dramatically enhanced BBB permeability to various molecular weight dextrans in primary HBMEC or hCMEC/D3 cultures, following incubation for 3 or 6 h. These BBB changes including the significant downregulation of ZO-1 expression at intercellular (TJs) clearly mirror what has been observed in vivo in animal studies that suggest the presence of amyloid beta is a factor in the “breach” of the BBB [44]. In experimental animals and cell cultures, beta amyloid proteins induce matrix metalloproteinase-9 (MMP-9) [45]. Transgenic mutant mice with mutant human amyloid protein show disruption of the BBB [46]. Furthermore, transgenic mice with apoE4 demonstrate BBB breakdown by activating the proinflammatory cyclophilin A-MMP-9 pathway in the brain pericytes, which, in turn, results in degradation of the BBB TJs and basal lamina (BL) proteins [47]. Mice overexpressing Aβ-precursor protein have pericyte loss, elevated brain Aβ40 and Aβ42 levels accelerating amyloid angiopathy, and cerebral amyloidosis [48].

**Fig. 5:**
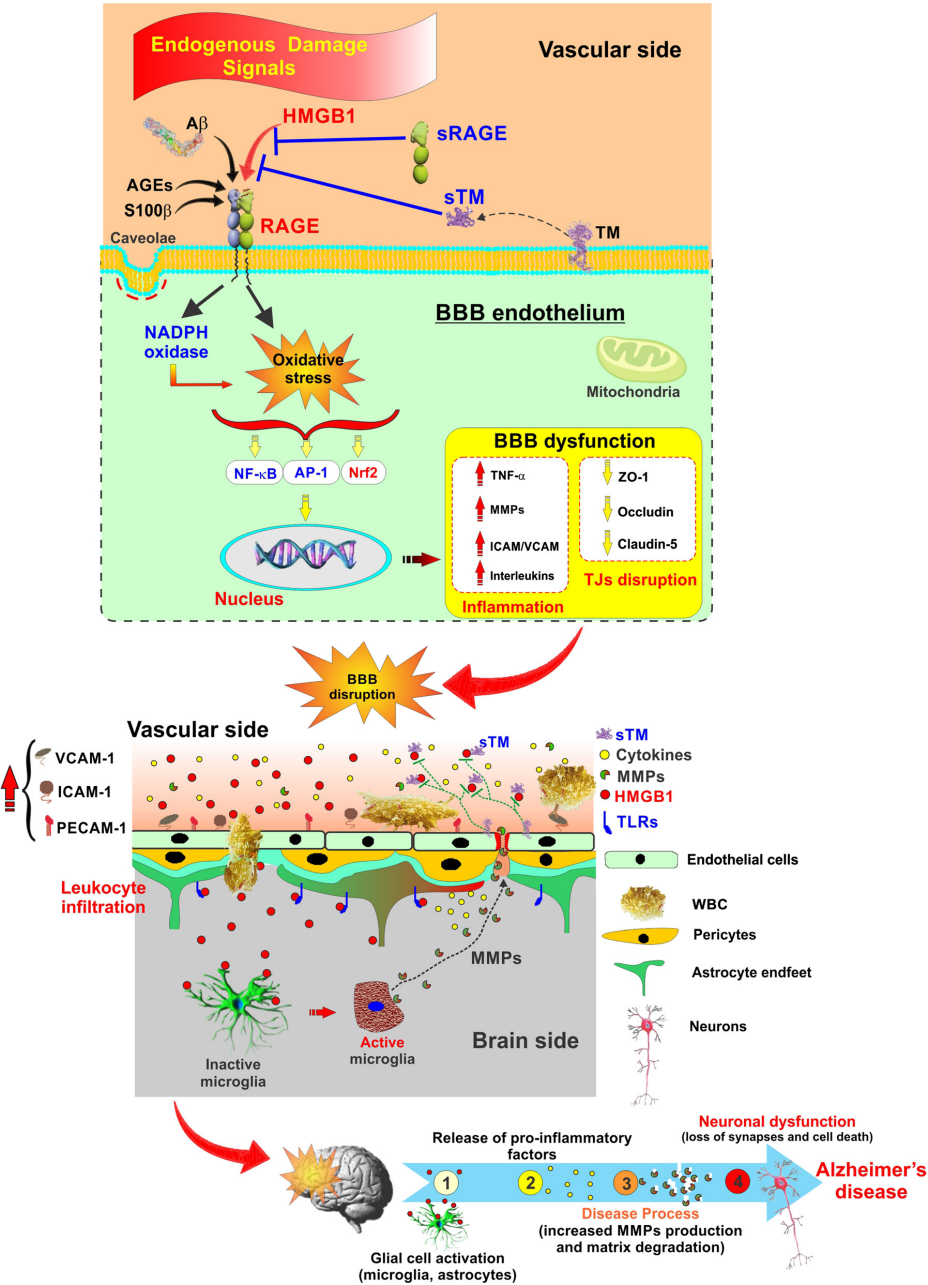
Schematic illustration of proinflammatory Aβ, DAMPs (HMGB1/S100B), and sRAGE acting at the BBB endothelium. Representation of BBB as *source* and *target* of neuroinflammation in AD. Endogenous damage molecules, DAMPs as well as Aβ, activate respective receptors to ramp up neuroinflammation. Soluble receptors such as sRAGE and sTM can neutralize some of these, and some may function as distinct biomarkers in progression of neurodegeneration in AD

Others have also shown that BBB dysfunction in rats, whether due to experimental stroke or traumatic brain injury (TBI), can be prevented by a neutralizing antibody to HMGB1 [49, 50]. Outside the cell, oxidized HMGB1 is known to ligate three different pattern recognition receptors all expressed on the surface of these ECs (as illustrated in Fig. 5). These include TLR2, TLR4, and RAGE [51, 52], and each can bind other ligands, such as Aβ, contributing to vascular complications of other diseases [53]. Both TLR and RAGE signaling leads to downstream NFkB activation with subsequent increase in the expression/release of tumor necrosis factor (TNF), thus ensuring that the inflammatory signal is maintained and amplified [54].

Over the last 15 years, the concept that the BBB might function both as *source* and *target* of proinflammatory factors is supported by numerous studies showing that the cerebral microcirculation is in an “activated proinflammatory” stage in neurodegenerative diseases such as AD [15, 55] and is a *target* for various proinflammatory factors. PAUSE Circulating Aβ is a proinflammatory cytokine-induced DAMP that binds to RAGE and TLR2 [56]. Like S100β and HMGB1, Aβ can exacerbate proinflammatory cytokine production such as TNF and interleukin-1 beta (IL-1β); however, in innate immunity, TNF dominates. When HMGB1 binds to RAGE and/or TLRs on microvascular ECs, it releases TNF and other cytokines [37], making the BBB also a *source* of these proinflammatory factors, and the axis in AD has attracted considerable attention [56]. Soluble RAGE or sRAGE, sometimes referred to as endogenous secretory RAGE, which can be produced either by splicing or proteolytic shedding, appears to play the role of a “decoy” to tie up RAGE ligands, such as S100/calgranulin family members, HMGB1, or Aβ [39]. The S100B/RAGE axis has been of considerable interest in AD, but earlier reports suggested that lower sRAGE levels characterized AD [39]. Interestingly, we found significant correlation between Aβ and HMGB1 or sRAGE (Fig. 1). Furthermore, the levels of HMGB1 in serum were in the range of rHMGB1 used in the BBB permeability experiments (Figs. 3a and 4), suggesting a continuum from normal through MCI to AD may exist. We did not find an indication that S100B might be useful, although a trend of significance in the MCI due to AD cohort was found (Fig. 1).

Just in the last decade, it has become increasingly appreciated that inflammation and coagulation are linked evolutionary defense systems [41], a fact that is slowly becoming recognized in the CNS as well. TBI and ischemic and hemorrhagic stroke are all characterized by increased levels of intraparenchymal thrombin and HMGB1 as well as evidence of BBB dysfunction [42, 49, 50]. In the brain, low concentrations of thrombin act through its principal receptor, PAR1, to induce neuroprotection [57]. In contrast, at higher concentrations, thrombin causes brain damage [58], where we showed that it appears to act via PAR4 [59–61]. Through PAR activation, thrombin directly affects the activity of multiple cell types and regulates a variety of biological functions, including inflammation, leukocyte migration, and vascular permeability [61]. Relevant to this and our current results, all PARs are expressed in microvascular ECs [62], and PAR1, at least, is expressed in ECs of the BBB [63].

Also attesting to evolutionary linkage, direct relationships also exist between thrombin and HMGB1. HMGB1 is involved in a number of systemic vascular diseases [64], is increased in stroke [65], and found around amyloid plaques and NFTs in AD brains [66], where thrombin and prothrombin accumulate as well [67]. Both HMGB1 and thrombin are released in various neurologic conditions, and HMGB1 promotes coagulation [68]. Taken together, these observations raise the possibility that HMGB1 and thrombin also participate during innate immune neuroinflammatory situations such as in AD and which contribute to BBB dysfunction and WBC transendothelial migration.

Finally, we have envisioned our present data in corroboration with previous findings schematically in Fig. 5. This shows that increased levels of danger/damage-associated, proinflammatory molecules (the alarmin DAMPs, HMGB1 and S100B, as well as Aβ) feature prominently in the AD continuum from normal aging through MCI by acting on TLRs and RAGE to induce neuroinflammatory signaling at the BBB endothelium. This results in increased expression of the transcription factor NFkB causing increased TNF and IL-1β (other interleukins), ROS, and adhesion molecules along with degradation of tight junction (TJ) proteins such as ZO-1. Both endogenous as well as exogenous means exist to combat this imbalance including the proteolytic release of sRAGE and sTM, both of which selectively bind up HMGB1 (possibly other alarmins) preventing engagement of RAGE or TLRs. This also allows for the development of novel therapeutic targets that act like sRAGE and sTM, in a manner less burdensome and potentially with fewer side effects than by administering neutralizing antibodies to HMGB1, such as recently used by others in rat models of stroke, TBI, and Parkinson’s disease [49, 50, 69].

## Conclusions

An increased understanding of the role of HMGB1 and thrombin in BBB dysfunction as well as activation and transendothelial migration of WBCs contributing to neuroinflammation in AD may allow for the discovery of novel therapeutic targets and treatment strategies. Not only might these facilitate treatment to halt progression in AD but these might also aid in means to detect the conversion from MCI to pre-clinical AD, as well as in other neurological disorders that display BBB dysfunction.

## Abbreviations

ANOVA: Analysis of variance
AD: Alzheimer’s disease
Aβ: Amyloid beta
BBB: Blood-brain barrier
DAMPs: Damage-associated molecular patterns
EC: Endothelial cell
ELISA: Enzyme-linked immunosorbent assay
hCMEC/D3: Human cerebromicrovascular endothelial cell line
HMGB1: High-mobility group box protein 1
HBMEC: Primary human brain microvascular endothelial cells
IL-1β: Interleukin-1 beta
MCI: Mild cognitive impairment
NFT: Neurofibrillary tangles
NVU: Neurovascular unit
PAR: Protease-activated receptor
RITC: Rhodamine B isothiocyanate
sRAGE: Soluble receptor for advanced glycation end products
TJ: Tight junction
TLR: Toll-like receptor
TM: Thrombomodulin
TNF: Tumor necrosis factor
WBCs: White blood cells
ZO-1: Zona occludins
